# A deprescribing medication program to evaluate falls in older adults: methods for a randomized pragmatic clinical trial

**DOI:** 10.1186/s13063-022-06164-5

**Published:** 2022-04-04

**Authors:** Joshua Niznik, Stefanie P. Ferreri, Lori Armistead, Benjamin Urick, Mary-Haston Vest, Liang Zhao, Tamera Hughes, J. Marvin McBride, Jan Busby-Whitehead

**Affiliations:** 1 Division of Geriatric Medicine and Center for Aging and Health, University of North Carolina at Chapel Hill, School of Medicine, Chapel Hill, NC USA; 2 Division of Pharmaceutical Outcomes and Policy, University of North Carolina at Chapel Hill, Eshelman School of Pharmacy, Chapel Hill, NC USA; 3 Center for Health Equity Research and Promotion, Veterans Affairs (VA) Pittsburgh Healthcare System, Pittsburgh, PA USA; 4 Division of Practice Advancement and Clinical Education, University of North Carolina at Chapel Hill, Eshelman School of Pharmacy, Chapel Hill, NC USA; 5 UNC Health, Chapel Hill, NC USA

**Keywords:** Pharmacist, Deprescribing, Opioids, Benzodiazepines, Primary care, Older adults, Falls

## Abstract

**Background:**

Opioids and benzodiazepines (BZDs) are some of the most commonly prescribed medications that contribute to falls in older adults. These medications are challenging to appropriately prescribe and monitor, with little guidance on safe prescribing of these medications for older patients. Only a handful of small studies have evaluated whether reducing opioid and BZD use through deprescribing has a positive impact on outcomes. Leveraging the strengths of a large health system, we evaluated the impact of a targeted consultant pharmacist intervention to deprescribe opioids and BZDs for older adults seen in primary care practices in North Carolina.

**Methods:**

We developed a toolkit and process for deprescribing opioids and BZDs in older adults based on a literature review and guidance from an interprofessional team of pharmacists, geriatricians, and investigators. A total of fifteen primary care practices have been randomized to receive the targeted consultant pharmacist service (*n* = 8) or usual care (*n* = 7). The intervention consists of several components: (1) weekly automated reports to identify chronic users of opioids and BZDs, (2) clinical pharmacist medication review, and (3) recommendations for deprescribing and/or alternate therapies routed to prescribers through the electronic health record. We will collect data for all patients presenting one of the primary care clinics who meet the criteria for chronic use of opioids and/or BZDs, based on their prescription order history. We will use the year prior to evaluate baseline medication exposures using morphine milligram equivalents (MMEs) and diazepam milligram equivalents (DMEs). In the year following the intervention, we will evaluate changes in medication exposures and medication discontinuations between control and intervention clinics. Incident falls will be evaluated as a secondary outcome. To date, the study has enrolled 914 chronic opioid users and 1048 chronic BZD users. We anticipate that we will have 80% power to detect a 30% reduction in MMEs or DMEs.

**Discussion:**

This clinic randomized pragmatic trial will contribute valuable evidence regarding the impact of pharmacist interventions to reduce falls in older adults through deprescribing of opioids and BZDs in primary care settings.

**Trial registration:**

Clinicaltrials.govNCT04272671. Registered on February 17, 2020

**Supplementary Information:**

The online version contains supplementary material available at 10.1186/s13063-022-06164-5.

## Administrative information

Note: the numbers in curly brackets in this protocol refer to SPIRIT checklist item numbers. The order of the items has been modified to group similar items (see http://www.equator-network.org/reporting-guidelines/spirit-2013-statement-defining-standard-protocol-items-for-clinical-trials/).

**Table:** 

Title {1}	A Deprescribing Medication Program To Evaluate Falls In Older Adults: Methods For A Randomized Pragmatic Clinical Trial
Trial registration {2a and 2b}.	This trial was registered on clinicaltrials.gov on February 17, 2020 (NCT04272671 - https://clinicaltrials.gov/ct2/show/NCT04272671 ).
Protocol version {3}	Protocol version 1.0 as of 10/05/2022.
Funding {4}	This work was funded by the Centers for Disease Control and Prevention (CDC) under Cooperative Agreement (5U01CE002955). Dr. Niznik is supported by a career development award from the National Institutes on Aging (1K08AG071794).
Author details {5a}	Joshua Niznik, PharmD, PhD1,2,3 Stefanie Ferreri, PharmD4 Lori Armistead, MA, PharmD4 Benjamin Urick, PharmD, PhD4 Mary-Haston Vest, PharmD, MS, BCPS4,5 Liang Zhao, MS5 Tamera Hughes, PharmD, PhD4 J. Marvin McBride, MD1 Jan Busby-Whitehead, MD1 1. Division of Geriatric Medicine and Center for Aging and Health, University of North Carolina at Chapel Hill, School of Medicine, Chapel Hill, NC, USA 2. Division of Pharmaceutical Outcomes and Policy, University of North Carolina at Chapel Hill, Eshelman School of Pharmacy, Chapel Hill, NC, USA 3. Center for Health Equity Research and Promotion, Veterans Affairs (VA) Pittsburgh Healthcare System, Pittsburgh, PA, USA 4. Division of Practice Advancement and Clinical Education, University of North Carolina at Chapel Hill, Eshelman School of Pharmacy, Chapel Hill, NC, USA 5. UNC Health, Chapel Hill, NC, USA
Name and contact information for the trial sponsor {5b}	This work was sponsored by the University of North Carolina at Chapel Hill
Role of sponsor {5c}	The funding organization has no role in the study design, data collection and analysis, protocol preparation, or the decision to submit the protocol for publication.]

## Introduction

### Background and rationale {6a}

Medication-related adverse events occur in more than 15% of older adults in the USA, with approximately half of these being preventable [1]. Some of the most devastating and costly adverse events among older adults are falls and fall-related injuries. A number of modifiable factors can influence the risk for falls among older adults [2], including medications that act on the central nervous system (CNS). These medications are often viewed as culpable due to side effects of dizziness, somnolence, and poor balance in older adults. A recent systematic review [3] found that more than two-thirds of older adults were prescribed fall-risk-increasing drugs (FRIDs) at the time of a fall-related injury. Among these, opioids and benzodiazepines (BZDs) were some of the most commonly prescribed FRID classes that may have contributed to falls.

Opioids and BZDs are CNS-active medications that are challenging to appropriately prescribe and monitor, yet are commonly used in older adults [4, 5]. Although these medications are frequently used in the treatment of pain, anxiety, and insomnia, they have also been shown to be associated with injurious falls in older adults, especially when used concurrently [3, 6, 7]. Even usual doses of these medications can be unsafe when prescribed in older adults due to altered drug pharmacokinetics and pharmacodynamics that occur as a consequence of physiologic aging [8]. A number of guidelines caution against the use of opioids and BZDs in older adults [9, 10], but offer little guidance on safe prescribing of these medications.

Efforts have been made by pharmacists to optimize medication use and reduce the risk for otherwise preventable medication-related falls through deprescribing [11–15]. Deprescribing is the supervised process of gradually reducing or discontinuing medications that may cause more harm than benefit [16]. Several studies have evaluated the effectiveness of targeted pharmacist-involved interventions to deprescribe BZDs in older adults, but few have addressed opioids in this population [17]. Interventions have included patient- or provider-targeted educational materials, electronic pharmacist communications, and pharmacist-led deprescribing services, all with varying degrees of success. Prior studies are limited in that few have addressed the use of both opioids and BZDs [18, 19], few have examined clinical outcomes beyond changes in prescribing, and many were conducted in small samples using non-randomized designs. Scalability of interventions may be a roadblock to larger evaluation studies. Models of care delivery that leverage the use of interventions delivered by a centralized team via health information technologies (i.e., EHRs) may be well-positioned to overcome this challenge.

Leveraging the strengths of a large academic health system and an existing centralized pharmacist care team, we are implementing a clinic randomized pragmatic trial to evaluate the impact of a targeted consultant pharmacist intervention delivered at multiple primary care practices to deprescribe opioids and BZDs and reduce falls among older adults.

### Objectives {7}

Our specific aims are (1) to use electronic health record (EHR) data to inform primary care providers of older adult patients who are at high risk of falls due to chronic use of opioids and/or BZDs and (2) to evaluate the impact of a targeted consultant pharmacist intervention on reducing opioid and BZD exposures as well as the rate of falls for older adults. This study will address a major gap in knowledge regarding the impact of centralized and targeted pharmacist services to improve outcomes in older adults through optimizing medication use.

### Trial design {8}

**Fig. 1:**
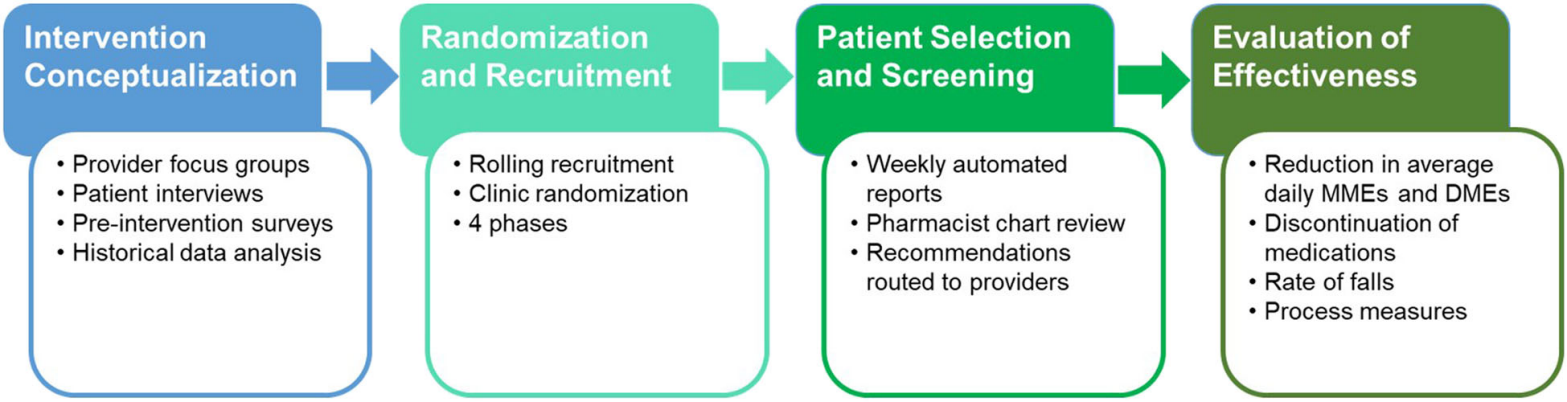
Study Design and Overview

This study is a pragmatic, randomized, clinical, exploratory trial of a consultant pharmacist intervention to reduce falls in older adults through deprescribing opioids and BZDs in primary care practices (Fig. 1). Primary care practices were randomized 1:1 to receive the consultant pharmacist intervention and followed for a 1-year period to evaluate changes in average daily morphine milligram equivalents (MMEs) and diazepam milligram equivalents (DMEs) as well as changes in the rate of falls, based on self-report. All study data is being collected through the UNC Health System’s EHR, Epic (Epic Systems Corporation, Verona WI).

## Methods: participants, interventions, and outcomes

### Study setting {9}

Clinics participating in the study are affiliated with UNC Physicians Network (UNC-PN) of the UNC Health System (UNC Health). UNC Health is a public, academic medical center operated by and for the people of North Carolina, serving patients from all North Carolina counties and throughout the Southeast United States. The UNC Physicians Network consists of more than 90 outpatient primary care practices and more than 300 providers. Practices are located in 14 counties throughout North Carolina and serve patients from both rural and urban areas of the state. All UNC-PN practices and UNC Health facilities use the same linked EHR (Epic), which served as the primary source of data for the study.

### Eligibility criteria {10}

**Fig. 2:**
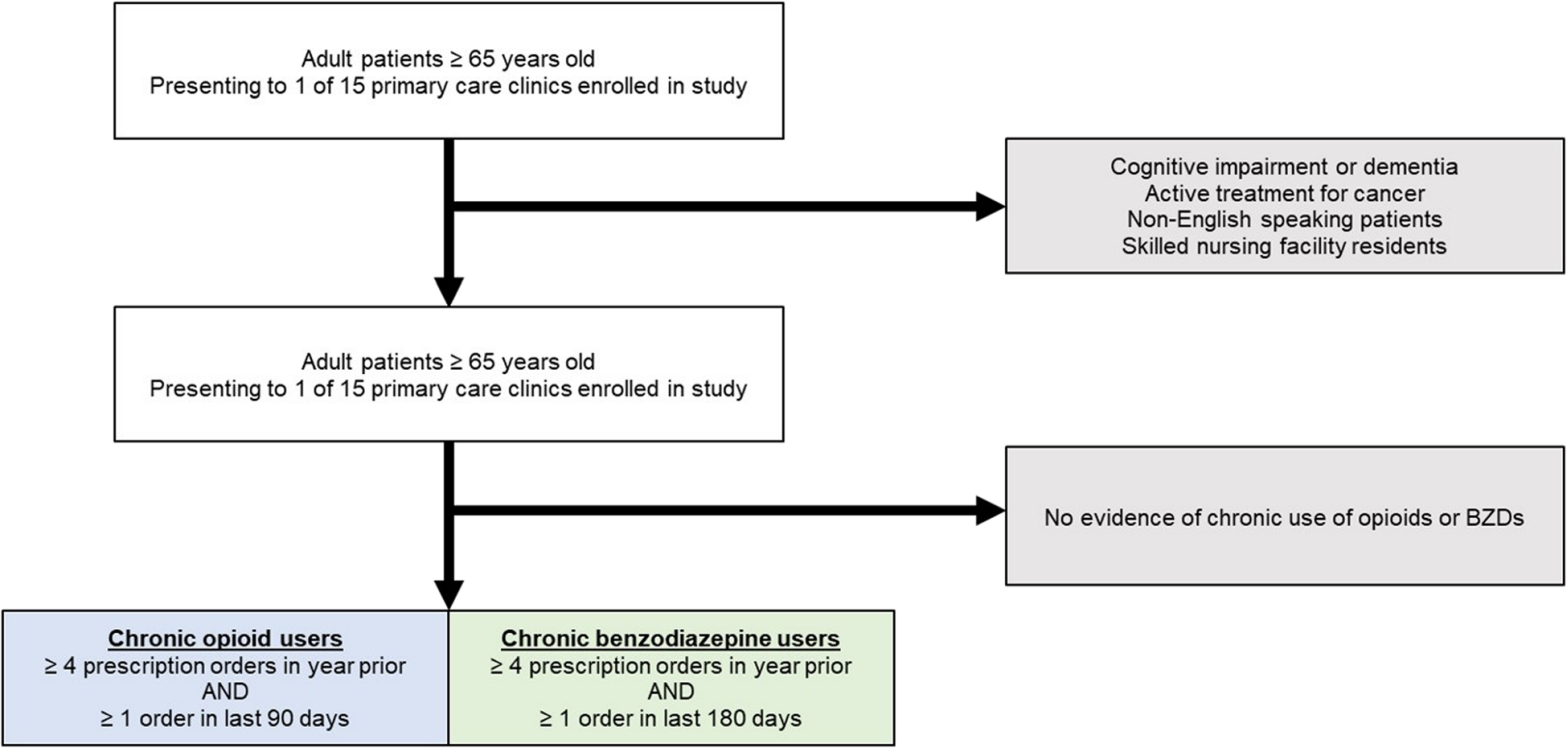
Inclusion and Exclusion Criteria

The target sample for the study was adults age 65 and older seen in primary care practices (Fig. 2). Patients with cognitive impairment or dementia were excluded based on the chronic conditions data warehouse (CDW) algorithm for identifying dementia [20]. We also excluded patients for whom chronic use of opioids or BZDs may be appropriate, making them ineligible for deprescribing. This included those actively receiving treatment for cancer, defined as having received chemotherapy or radiation therapy in the past year based on treatment orders in the EHR, and/or having received a surgical procedure for a cancer diagnosis based on the International Classification of Diseases, Tenth Revision (ICD-10) codes. Finally, non-English-speaking patients and individuals residing in nursing facilities were excluded.

Among eligible older adults, we identified those with evidence of chronic use of opioids or BZDs. There is limited and inconsistent guidance from the literature regarding what qualifies as “chronic use.” Some definitions rely on the frequency of fills or proportion of days covered (PDC) while others rely on average daily milligram equivalents [21–26]. We were only able to obtain data on medication orders, meaning that our definition for chronic use had to be defined based on a certain number of orders issued over a confined time period.

In our original protocol, our definition for chronic use required at least 2 prescriptions for opioids or BZDs, respectively, over a 1-year period. After several months of piloting the intervention, preliminary data collected by the clinical pharmacy team suggested that our definition for chronic use was too broad. Nearly two-thirds of patients identified by our definition were being ruled out as ineligible for deprescribing by the clinical pharmacists. We hypothesized that the clinical judgement of the pharmacists was based on the consistency of prescriptions and whether prescriptions were recent. Using pharmacists’ identification of patients as chronic users as the gold standard, we evaluated the sensitivity and specificity for several alternate definitions for chronic use based on fill frequency and the recency of the last fill to the end of the evaluation period.

We used prescription orders from the initial phase of the study to conduct a series of logistic regression models using each definition to predict inclusion for eligibility based on documented adjudication data from the CAMP pharmacy team. The results of our analyses are presented in Table S1. Based on our findings, we revised our definitions with the goal of improving specificity, but still favoring sensitivity in order to maximize the pool of eligible patients. Chronic use of opioids was revised to at least four prescriptions in the prior year with at least one prescription in the last 90 days (sensitivity: 88.9%, specificity: 64.8%). Chronic use of BZDs was revised to at least four prescriptions in the prior year with at least one in the last 180 days (sensitivity: 81.8%, specificity: 48.7%). After 2 weeks of testing these definitions, we observed a substantial decrease in the number of patients excluded by clinical pharmacists due to non-chronic use.

### Who will take informed consent? {26a}

Each clinic designated a “study champion” (practice manager or medical director) who signed a consent form prior to the clinic’s participation. Patients within participating clinics are secondary subjects of the study and will all receive the same standard of care as provided by their clinic. Therefore, written consent will not be requested from individual patients within participating clinics. However, clinics were provided with an information sheet for display to let their patients know that the clinic is participating in this study.

### Additional consent provisions for collection and use of participant data and biological specimens {26b}

Study champions agreed to have clinic data used even if they choose to withdraw from the trial. Participants also agreed to have relevant data shared with the funding sponsor and the practice network to which the clinics belong. This trial does not involve collecting biological specimens.

## Interventions

### Explanation for the choice of comparators {6b}

As this was a pragmatic trial, control clinics received usual care from their primary care providers. Providers in control clinics were provided with educational materials related to immunizations to distribute to patients. The same data is collected for control and intervention clinics each week, but these are only provided to the research team.

### Intervention description {11a}

The intervention for this study was conceptualized as a targeted consultant pharmacist model [27]. Pharmacist-physician collaborations are not a new concept. However, these collaborations often have limited impact due to information accessibility and communication barriers for community pharmacists or limited sustainability due to the costs of maintaining pharmacist services embedded in outpatient clinics. Alternative models of care that can overcome these limitations are greatly needed. A model of care that utilizes a centralized consultant pharmacist team and leverages the resources and interoperability of practices in a large health system has the potential to overcome these limitations [28, 29]. In this model, the pharmacist team is external to the practice, but still has access to the EHR data for all clinics in the system. Consultant pharmacists can conduct targeted medication reviews for specific drug classes or chronic medical conditions and then provide individualized recommendations for multiple clinics simultaneously, thereby improving overall efficiency.

We developed a toolkit and process for deprescribing opioids and BZDs in older adults based on our study team’s prior work [12, 15] and with input from our interprofessional team of pharmacists, geriatricians, and investigators. The process, entitled A-TAPER (Fig. S1), includes Assessing medication use, Talking about risks and benefits, selecting Alternatives, Planning nest steps, Engaging patients, and Reducing doses. The toolkit itself is an online repository that contains both provider- and patient-facing educational materials for deprescribing, alternative and adjunctive therapies, and monitoring of opioids and BZDs (https://deprescribe.web.unc.edu/). The A-TAPER process is highly adaptable to any class of medications, while still maintaining an individualized approach to deprescribing and served as the foundation on which the intervention for this study was developed.

The consultant pharmacist intervention for the study was designed in collaboration with the Carolina Assessment of Medications Program (CAMP) team. The CAMP team provides pharmacist-led consultant comprehensive medication management (CMM), as well as targeted disease state interventions including a Diabetes Management Service, Hypertension Management Service, Transitions of Care Service, Chronic Obstructive Pulmonary Disease Service, and Anticoagulation Management. The CAMP team is comprised of pharmacists, pharmacy technicians, and schedulers and aims to improve quality of care, control health care costs, and work in partnership with UNC Health providers to deliver coordinated care to UNC Health patients.

The Opioid & Benzodiazepine De-escalation Consult Service offered through the CAMP team serves as the intervention for the study. Weekly automated reports identify patients meeting inclusion criteria within an intervention clinic (described above), with a scheduled appointment in the upcoming week (Fig. S2). These reports are queried from the organization’s data warehouse and include EHR data. Patient reports are sent to a secured shared drive accessible to the CAMP pharmacists and include patient name, medical record number (MRN), provider name, active opioid and/or BZD therapy, and prescription information. The CAMP pharmacists then review the patient’s chart within the EHR, as well as the state prescription drug monitoring program (PDMP), and make recommendations for a taper as appropriate, using the A-TAPER pathway (described above).

The research team partnered with the EHR development team to develop discrete documentation (i.e., smart phrases) which allowed for data extraction and monitoring of de-escalation recommendations, initial or follow-up recommendation from the pharmacist, rationale if no de-escalation of therapy was recommended. Recommendations are sent directly through the EHR to the appointment provider, as well as documented in a Progress Note. If clarification is needed, the appointment provider can send messages to the CAMP pharmacist directly, which are not saved within the EHR. Intervention clinics were also provided with targeted training videos and other educational materials on falls risk and deprescribing as well as patient educational materials from the Centers for Disease Control and Prevention (CDC) Stopping Elderly Accidents, Deaths, & Injuries (STEADI) initiative to distribute in their clinic.

### Criteria for discontinuing or modifying allocated interventions {11b}

Any clinic wishing to withdraw from the intervention group can do so at any time. Providers and patients also reserve the right to opt out of any pharmacist recommendations for changing medication therapy.

### Strategies to improve adherence to interventions {11c}

A postdoctoral research fellow will conduct monthly check-ins to evaluate provider satisfaction with the intervention and to identify potential issues regarding non-adherence intervention to bring to the study team.

### Relevant concomitant care permitted or prohibited during the trial {11d}

As this was a clinic-level intervention, patients were free to seek any concomitant care during the trial. Patients in control and intervention clinics will be permitted to receive usual care from their provider. Potential protocol deviations, such as switching clinics, will be addressed in secondary per-protocol analyses.

### Provisions for post-trial care {30}

Prescribers of these medicines are familiar with potential adverse events. Educational materials will continue to be available to prescribers following the intervention. Usual care will continue to be available to assist patients if the need arises post-intervention.

### Outcomes {12}

Our primary outcome is the change in exposure to opioids and BZDs, as measured by dosage reductions and/or discontinuations. Medication exposures will be operationalized as average daily MMEs for opioids and average daily DMEs for BZDs. Adapting methodology from prior studies using prescription data to identify deprescribing [30–32], we will evaluate several gap lengths ranging from 30 to 90 days. We will also explore the use of “smart phrase” data (described above) to adjudicate discontinuation events. A secondary outcome is the occurrence of falls as measured by patient responses to CDC STEADI questions integrated in the EHR, using the response from the latest clinic visit after the index date.

Other secondary outcomes include prescriber knowledge, attitudes, and perceived self-efficacy in deprescribing opioids and BZDs in older adults. Prior to launching the intervention, a survey was disseminated to all providers in the 15 participating clinics. The survey was a modified version of that developed by Farrell and colleagues [33]. Data will be collected post-intervention to assess whether the deprescribing toolkit and consultant pharmacist recommendations changed providers’ responses from pre- to post-intervention.

### Participant timeline {13}

The intervention will be conducted from May 2020 through October 2021, with each clinic having at least 32 weeks and up to 52 weeks to participate (Fig. S3). Individual patients will be indexed into the study on the date of their first visit to one of the participating clinics during the intervention period. Pre-intervention data will be collected using EHR data from the year prior to the patient’s index date. Post-intervention data will be collected for up to 1 year following each patient’s index date.

### Sample size {14}

We chose to calculate power for our primary analyses only. Power calculations assume 8 clusters (clinics) in the intervention arm versus 7 clinics in the control arm in a parallel cluster randomized trial pretest-posttest trial design. Because each patient (barring dropout) has both baseline and post-intervention level of exposure, an analysis of the covariance model will be fitted to the outcomes where the outcome at pretest (baseline) is the covariate.

Power calculations are presented in Tables S2 and S3. Sample size calculations were performed using nQuery 7.0 and take into account unequal cluster sizes summarized by the coefficient of variation (CV), which is equal to the standard deviation (SD) of cluster size (in terms of the number of data points from the clinic) divided by the mean cluster size. In the power calculation, we assume a mean cluster size of 60 for each outcome, and a CV = 0.70. We hypothesize a value of the multiple correlation coefficient *R* = 0.7 in our computations of covariate-adjusted power. For the primary outcomes of opioid and BZD exposure (MME and DME, respectively), power is based on the natural log transform which is needed to achieve approximate normality. Therefore, the effect size used in power calculations is for the between-group difference in log means; however, its equivalent (upon exponentiation), the fold change based on the original scale of the outcomes, is also reported in the tables to enhance the interpretation of results. Power analysis was informed by historical data from 1 year prior to study recruitment.

Unadjusted for clustering, the mean log MME (SD) was 2.50 (1.15) based on *N* = 961 observations, and the mean log DME (SD) was 1.54 (1.06) based on *N* = 959. A preliminary generalized estimating equations (GEE) analysis (no covariates, identify link, normal distribution, exchangeable correlation) of pretest data was performed for MME and DME on the natural log scale. The number of clusters (practices) was 15 in each model, and cluster sizes varied widely (8–187 in the log MME analysis and 16–128 for the log DME analysis). The model-based results of GEE were similar to observed data: The mean log MME was 2.37 (1.15) with ICC = .063; the mean log DME was 1.53 (1.06) with ICC = 0.019. Power to detect a difference between intervention and control treatment arms is very sensitive to (i) the intraclass correlation (ICC; 0.01 to 0.06), (ii) magnitude of fold change (10 to 40%) between treatments, and (iii) between unadjusted and covariate-adjusted models.

Under reasonable assumptions for effect size and ICC estimates, we find that we will have at least 80% power to detect a 30% reduction in MMEs or DMEs in most scenarios. If the effect of the intervention is small, or clinic-to-clinic variation is quite large relative to patient-level variation, this study may be underpowered. However, we view this as unlikely, given the analysis of existing data which support ICCs less than 0.02 and literature which finds similar interventions frequently achieve reductions on MME and DME in excess of 30% [34–37].

### Recruitment {15}

Clinics were recruited in phases into the study beginning in December 2019 and ending in December 2020. Using historical data from the UNC Carolina Data Warehouse (CDW), we were able to identify the approximate number of eligible patients at all UNCPN clinics. Clinics were targeted on the basis of the number of eligible patients and geographic representation of the state. We aimed to recruit between 15 and 20 primary care clinics to our study, based on preliminary power calculations using the historical clinic data. After recruiting 15 clinics, revised power calculations suggested that a sufficient sample size had been achieved and recruitment ended.

## Assignment of interventions: allocation

### Sequence generation {16a}

Recruitment and randomization were conducted in three phases, with at least four clinics in each phase, so as to not delay the rollout of the intervention. After each phase of recruitment, a series of pseudo-random numbers generated by SAS (SAS Institute, Cary, NC) was used to randomly assign clinics to the control or intervention arms on a 1:1 basis.

### Concealment mechanism {16b}

Not applicable—clinics and providers were not blinded.

### Implementation {16c}

Clinics in each recruitment phase were randomized separately (i.e., block randomization) due to different enrollment periods, and in the one phase where the number of clinics was uneven, two clinics were assigned to intervention and one was assigned to control. As a result, eight clinics were randomized to the intervention with seven serving as controls.

## Assignment of interventions: blinding

### Who will be blinded {17a}

Clinics and providers were not blinded. The study team provided a letter of invitation to participate in the study and met with clinic providers and staff in person to discuss any questions about the nature of the study, procedures, risks, and benefits. A copy of the study’s IRB application was provided when requested.

### Procedure for unblinding if needed {17b}

The design is an open label with only data analysts being blinded so unblinding will not occur.

## Data collection and management

### Plans for assessment and collection of outcomes {18a}

On a weekly basis, a report is generated for the research study team that includes all patients who meet inclusion criteria and presented at one of the participating study clinics (control and intervention). Data elements in this report include clinic location, BZD and/or opioid medication order, originating clinic for prescription, order date, dose, quantity, days’ supply, refills authorized, ordering provider name, ordering provider identifier, STEADI question responses, age, gender, payor status, race, co-morbid conditions, visit type/reason, concomitant medication list, and whether an annual wellness visit was completed in the past year. These weekly reports serve as the main source of data for analysis. Data is also collected from CAMP pharmacist notes using “smart phrases”, which are stored as standardized data elements for extraction. These data elements provide details on whether patients were opted out of the intervention by the pharmacist or prescriber, number of opioid and BZD tapering recommendations, recommendations for adjunct or alternate therapy, and completion of recommended tapers.

Calculating medication exposures involves combining several fields of information from medication orders to calculate MMEs and DMEs. Two student pharmacists (SH, SM) review order directions to extract the daily number of units and discrepancies were resolved by a pharmacist (JN, BU). Daily units are divided by the total quantity issued to determine the intended days’ supply of each order. Daily units are then multiplied by the prescribed dose from the medication name to obtain the daily milligram exposure which was subsequently converted to average daily MMEs or DMEs. We assume that medication orders are filled on the date written unless the supply overlapped with another medication of the same type. In these cases, the fill date is adjusted to be the day following the final day the patient was expected to have supply on hand. Supply carrying over the end of the period was truncated. To calculate a total daily sum, the sum of each individual opioid or BZD is summed for each day. Total exposure over the 1-year period is calculated as the sum of the average daily MMEs or DMEs times the days’ supply for each order divided by 365 days. As an alternate measure of changes in medication exposure, we will evaluate the rate of complete discontinuation, defined based on gaps in estimated medication supply.

A secondary outcome is the occurrence of falls as measured by patient responses to CDC STEADI questions integrated in the EHR, using the response from the latest clinic visit after the index date.

### Plans to promote participant retention and complete follow-up {18b}

Clinic study champions and providers will be contacted by a postdoctoral scholar on a monthly basis to assess satisfaction, retention, and follow-up.

### Data management {19}

Data quality checks will be performed by the members of the data analysis subgroup upon receipt. Analysts will evaluate the range and distribution of patient index dates to verify that only visits occurring within the study time frame are included in analyses. Data from prescription orders will be reviewed by the team to ensure that patients meet our criteria for chronic use (described above) and that prescription orders fall within the study time frame (1 year prior to index date to 1 year following index date). After converting individual prescription orders into medication exposures, a member of the data analysis team will review the overall distribution of average daily MMEs and DMEs and hand verify calculations for a random sampling of prescription orders for accuracy.

### Confidentiality {27}

EHR data will be stored on a secured shared drive maintained by the North Carolina Translational and Clinical Sciences Institute (NCTraCS) with access restricted to the research team only. A secure research workstation with limited access for the study’s data analysis subgroup will be used for analysis. Identifiers will be removed using the HIPAA Safe Harbor method. Names will be removed, MRNs will be encrypted as random identifiers, and any dates will be shifted. Data will be maintained for the duration of the study and 1 year afterwards. One year following the study conclusion (when the IRB is closed out), NCTraCS will be notified to archive the data (the research team’s access will be removed). Archived data will be stored indefinitely.

### Plans for collection, laboratory evaluation, and storage of biological specimens for genetic or molecular analysis in this trial/future use {33}

No biologic specimens are being collected for this study.

## Statistical methods

### Statistical methods for primary and secondary outcomes {20a}

We will examine between-group differences in opioid and BZD exposure associated with an increased risk of falls in the intervention and control groups. Analyses will be performed using an intention-to-treat approach, and index dates for the intervention group will correspond to the date on which they first had an eligible clinic appointment during the intervention period. Index dates for otherwise eligible patients from the control group will be the first visit to a control clinic during the intervention period.

Our primary hypotheses are as follows: (1) relative to individuals in both control groups, individuals in both intervention groups will experience a significant reduction in the milligram equivalent exposure to opioids and BZDs during the 1-year follow-up period; (2) relative to individuals in both control groups, individuals in both intervention groups will experience a significantly greater discontinuation rate of opioids and BZDs during the follow-up period 181 to 360 days after index.

There are two overlapping groups of patients aged 65 and older that will be included in this study: chronic opioid users and BZD users. For each hypothesis, there will be separate models comparing the impact of the program on opioid users and BZD users. Patients who use both will appear in both models. To examine any differential impact of the program among patients who use both classes of medications, a sensitivity analysis will be performed and will include an indicator variable for patients who use both classes. Analysis of covariance (ANCOVA) will be used to estimate the effectiveness of the intervention. The dependent variable for these models will be MME or DME during the intervention period, with patient assignment to intervention or control group as the primary independent variable of interest. To account for differences in baseline MME and DME exposure, baseline MME/DME will be included as a fixed effect in the model. We will include independent variables known to be predictors of MME or DME exposure (described above) which may differ between intervention and control clinics. Covariates will be measured at the time of each patient’s index date. A log link with negative binomial distribution will be included to account for skew in the data. Finally, we will include a repeated measure for the clinic to account for clustering of patients within clinics. For analyses evaluating discontinuation of opioids or BZDs as the outcome, we will conduct logistic regression models to evaluate the odds of discontinuation between control and intervention groups.

Our secondary hypothesis is as follows: Relative to individuals in both control groups, individuals in both intervention groups will experience a reduction in the risk of experiencing a fall following the index date. Risk will be operationalized as the odds of experiencing a fall in the 12 months post-index compared to the 12 months pre-index. The models used for this analysis match those used for the primary analysis, with the exception that the models for fall risk will use a log link with a binomial distribution and not include baseline values.

### Interim analyses {21b}

Interim analyses will include descriptive summaries of eligible patients, including their socio-demographic characteristics and average daily medication exposures, reported as MMEs and DMEs. After enrollment has concluded, a summary of all enrolled patients and their descriptive characteristics will be presented to the study team. Clinic-level prescribing and demographic information may be provided to clinics, if requested. Given the low level of risk associated with this intervention, there are no plans to stop the intervention as a result of interim analyses.

### Methods for additional analyses (e.g., subgroup analyses) {20b, 20c}

Our primary analysis will use an intent-to-treat approach in which patients are allocated to control or intervention clinics based on their index visit. The same data will be collected for all patients, regardless of follow-up status or protocol adherence. Potential protocol deviation will be addressed in additional per-protocol analyses in which patients who received care at both intervention and control clinics will be excluded.

### Plans to give access to the full protocol, participant-level data, and statistical code {31c}

The full protocol may be requested at the permission of the principal investigators.

The datasets generated and/or analyzed during the current study are not publicly available as clinical data are the property of the healthcare system and its patients.

Reasonable data requests, including statistical code, may be considered by the authors with additional permission of the principal investigators, UNC Chapel Hill and its associated Institutional Review Board, and UNC Health

## Oversight and monitoring

### Composition of the coordinating center and trial steering committee {5d}

*Coordinating Center and Steering Committee: *Lori Armistead, MA, PharmD; Jan Busby-Whitehead, MD; Stefanie Ferreri, PharmD; Cristine Henage, EdD; 

Tamera Hughes, PharmD, PhD; Casey Kelley, MPH; Claire Larson, MD; Jason Liu, PharmD, MIDS; J. Marvin McBride, MD; Joshua Niznik, PharmD, PhD; John S. Preisser, PhD; Ellen Roberts, PhD, MPH; Kimberly Sanders, PharmD; Marina Snellings Stamopoulos, PharmD; Benjamin Urick, PharmD, PhD; Mary-Haston Vest, PharmD, MS, BCPS; Daniel Wolverton, PharmD; Ying Zhang; Liang Zhao, MS.

*Qualitative Data Working Group: *Lori Armistead, MA, PharmD; Jan Busby-Whitehead, MD; Stefanie Ferreri, PharmD; Cristine Henage, EdD; Tamera Hughes, PharmD, PhD; Casey Kelley, MPH; Joshua Niznik, PharmD, PhD; Ellen Roberts, PhD, MPH.

Data Analysis Working Group: Lori Armistead, MA, PharmD; Jan Busby-Whitehead, MD; Stefanie Ferreri, PharmD; Tamera Hughes, PharmD, PhD; Jason Liu, PharmD, MIDS; Joshua Niznik, PharmD, PhD; Benjamin Urick, PharmD, PhD; Mary-Haston Vest, PharmD, MS, BCPS; Daniel Wolverton, PharmD; Liang Zhao, MS.

*Toolkit Working Group: *Lori Armistead, MA, PharmD; Jan Busby-Whitehead, MD; Stefanie Ferreri, PharmD; Tamera Hughes, PharmD, PhD; Claire Larson, MD; J. Marvin McBride, MD; Kimberly Sanders, PharmD

### Composition of the data monitoring committee, its role, and reporting structure {21a}

A data monitoring committee was not deemed necessary by the Institutional Review Board of our institution as there are minimal safety concerns involved in this study. The Data Analysis Working Group (above) oversees data collection, storage, evaluation, and monitoring.

### Adverse event reporting and harms {22}

The primary mechanism for safety monitoring will be monthly check-ins by a postdoctoral research fellow who is part of the study team. As a part of monthly check-in calls, the fellow will inquire about any reported adverse effects from opioid and BZD deprescribing, such as increased pain, anxiety, and trouble sleeping. The educational intervention will include instructions for providers to notify clinic staff of any adverse effects, such as withdrawal. Additionally, the instructions will state that the provider should notify the study team directly if any serious adverse events, such as seizures from BZD, occur. Withdrawal symptoms are a normal and common result of deprescribing. Prescribers of these medicines are familiar with these symptoms, and additional information on withdrawal will also be included in the educational materials. Usual care will be available to assist patients if the need arises.

### Frequency and plans for auditing trial conduct {23}

The full study team and key stakeholders will continue to meet monthly to review progress updates, challenges, protocol adherence, and potential protocol deviations.

### Plans for communicating important protocol amendments to relevant parties (e.g., trial participants, ethical committees) {25}

The sponsor was notified of all protocol amendments in advance of study recruitment. Modified protocols were documented and approved with the IRB and the scientific review committee. The protocol was also updated in the clinical trial registry. The sponsor and IRB will be notified of any further modifications as relevant.

## Dissemination plans {31a}

The UNC study team will work with the CDC (funder) through the cooperative agreement to determine an appropriate dissemination plan. Findings will be presented at the national pharmacy and interprofessional geriatric meetings. Manuscripts based on our findings will be submitted to peer-reviewed journals in pharmacy, geriatrics, and internal medicine.

## Discussion

This study will contribute valuable evidence regarding the impact of pharmacist interventions to reduce falls in older adults through deprescribing of opioids and BZDs in primary care settings. The Pharmacy Quality Alliance (PQA) has identified the use of opioids and BZDs as priority areas for pharmacists to reduce harm and is currently developing quality measures to quantify potential inappropriate use of these medications [38]. 

To date, there have been few large randomized studies of pharmacist interventions targeting deprescribing of both opioids and BZDs [17]. Prior studies have shown that pharmacist interventions are effective in reducing the use of high-risk medications, but few large studies have examined whether this leads to meaningful reductions in important outcomes such as falls. The present study will address this gap in knowledge by examining the impact of a pharmacist-driven intervention on both prescribing and clinical outcomes. We will also be able to evaluate process measures as part of our study, by extracting detailed data from clinical pharmacy notes to evaluate actionable opportunities for deprescribing. This data will provide valuable insight into the feasibility of a targeted consultant pharmacist model delivered via the EHR as a means to improve prescribing and outcomes across multiple clinical sites within a large health system.

There are several limitations that must be considered in order to accurately interpret our potential findings. Our study is being conducted among multiple primary care practices, but within a single health system. Thus, our findings may not be generalizable to other health systems where standards of practice, policies, and norms are likely to differ. There is also a degree of heterogeneity at the clinic level that may influence the acceptability and success of a centralized clinical pharmacist service. The design and data sources of our study also have limitations. We acknowledge that our criteria for chronic opioid and BZD use are imperfect as we were limited to using prescription orders, rather than prescription refill data. It is possible that our criteria may over-estimate the prevalence of chronic use of opioids and BZDs among older adults seen at each practice, particularly given that we are unable to validate whether prescription orders were actually filled. Although prescription refill data would provide us with greater certainty that medications were being received, there is still the limitation that this may not accurately reflect how patients are actually taking medications on a daily basis. We are confident that using prescription orders will still yield a reasonable estimate of medication exposures. At the same time, we will not have access to prescription orders issued from prescribers external to clinics enrolled in the study, which may result in underestimation of medication exposures. However, given that the intervention is being delivered at the clinic level, we would not expect to see an effect on prescription orders for providers not participating in the intervention. Prescriber-level autonomy and heterogeneity may also affect the interpretability of our findings. Our intervention assumes that prescribers will act rationally based on recommendations provided by the clinical pharmacy team. It is possible that some prescribers may be more amenable to recommendations than others. We also acknowledge that our ability to identify falls using self-reported measures from the EHR may result in underreporting of outcomes, but we also intend to explore measures of health service utilization for falls using ICD-10 codes. Finally, we have outlined a number of independent variables to include in our analyses as potential confounders, to account for potential failure of randomization of clinics. However, we still acknowledge the potential for bias due to unmeasured confounding.

## Trial status

This trial was registered on clinicaltrials.gov on February 17, 2020 (NCT04272671—https://clinicaltrials.gov/ct2/show/NCT04272671). At the time of submission (October 2021), all 15 primary care clinics enrolled in the study have been randomized and the intervention has been implemented. Recruitment began in December 2019 and was completed in December 2020. For phase 1 clinics, the intervention began in May 2020 and completed in June 2021. For phase 2 clinics, the intervention began in August 2020 and completed in October 2021. For phase 3 and 4 clinics, the intervention began in January 2021 and completed in October 2021. Data collection for all enrolled subjects will continue through October 2022. Every effort was made to submit this protocol prior to the end of patient recruitment. The complexities of shifting this study during COVID did not allow the study team to submit prior to when the last patient was enrolled.

## Supplementary Information

**Additional file 1:** Supplementary Materials

## Abbreviations

BZDs: Benzodiazepines
DME: Diazepam milligram equivalent
HER: Electronic health record
MME: Morphine milligram equivalent
UNC: University of North Carolina
UNCPN: UNC Physicians Network
